# Supplementary material for: B. subtilis Sec and Srp Systems Show Dynamic Adaptations to Different Conditions of Protein Secretion
Source: Cells. 2024 Feb 22;13(5):377. doi: 10.3390/cells13050377 (PMC10930709; doi:10.3390/cells13050377)
Supplement: Supplementary file 1 [file cells-13-00377-s001.zip › cells-2832288-supplementary.pdf]

Fiedler and Graumann, 2024

Supplementary material

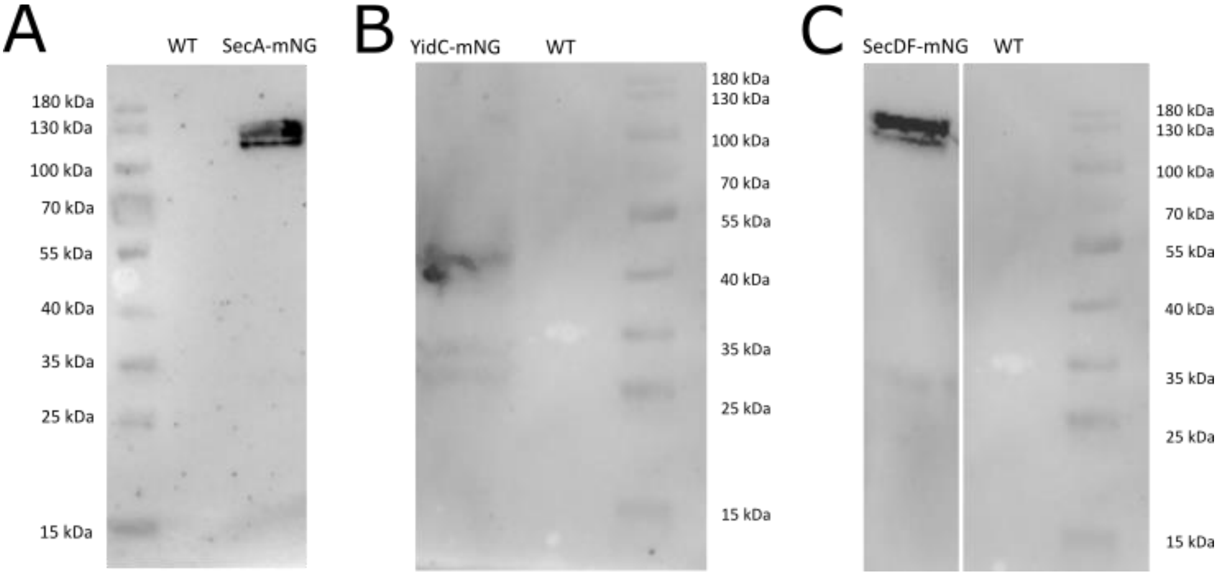

Figure S1: Western blots of strains expressing mNeongreen fusions as sole source of the respective protein. The first antibody, mNeongreenTag antibody (#55074, Cell Signaling Technology, Danvers, Massachusetts, USA), is the second anti-rabbit antibody. The wildtype (WT) is the negative control in all cases. A) western blot of SecA-mNG. The band size equals 122 kDa (95 kDa for SecA and 27 kDa for mNG). B) western blot of YidC-mNG. The height of the Band should be at 56 kDa (29 kDa for YidC and 27 kDa for mNG) but runs abnormally lower (not unusual for membrane proteins). C) Western blot for SecDF-mNG. The size of the fusion is 108 kDa (81 kDa for SecDF and 27 kDa for mNG).

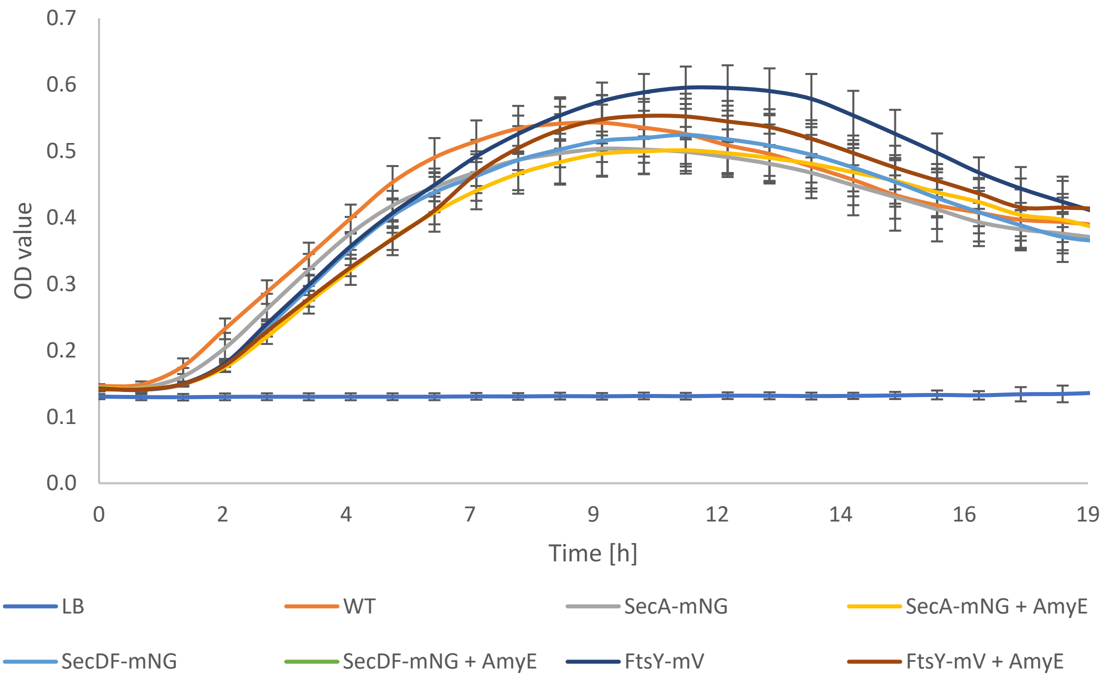

Figure S2: Growth curves of strains expressing C-terminal fusions to mNG to test for functionality. The growth curves were performed in 9 wells in parallel, in conditions where aeration is limiting, such that growth is less efficient than in open shaking flasks. Strains named with “+ AmyE” are the AmyE overproduction strain (containing pM11k_AmyE). All shown fusions are fusions of essential genes, and therefore, it can be said that all fusions are functional.

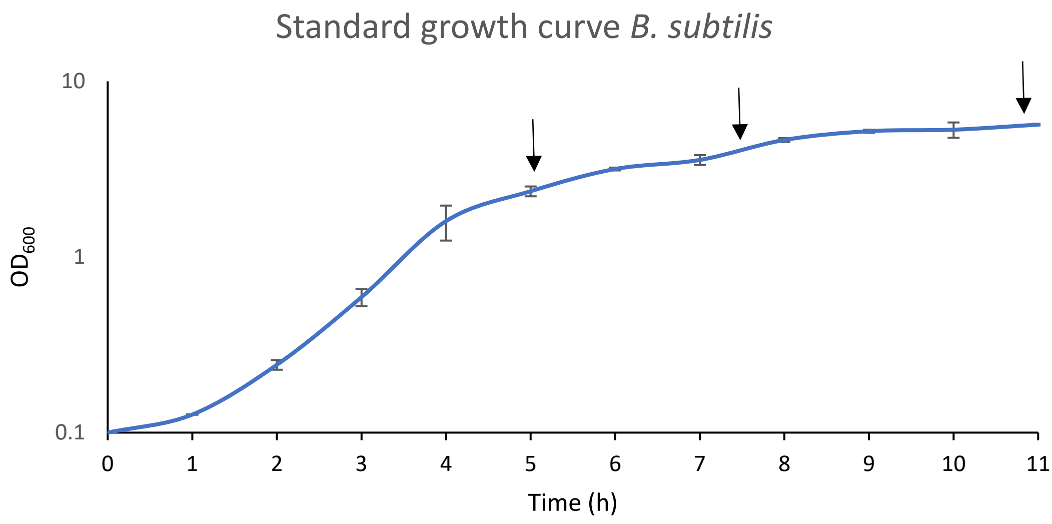

Figure S3: Standard growth curve of *B. subtilis* cells expressing SecA-mNG as the sole source of the protein under optimal growth conditions (shaking flask, rich medium). Arrows indicate time points in which SMT experiments were performed at optical densities of 2, 4, or 6.

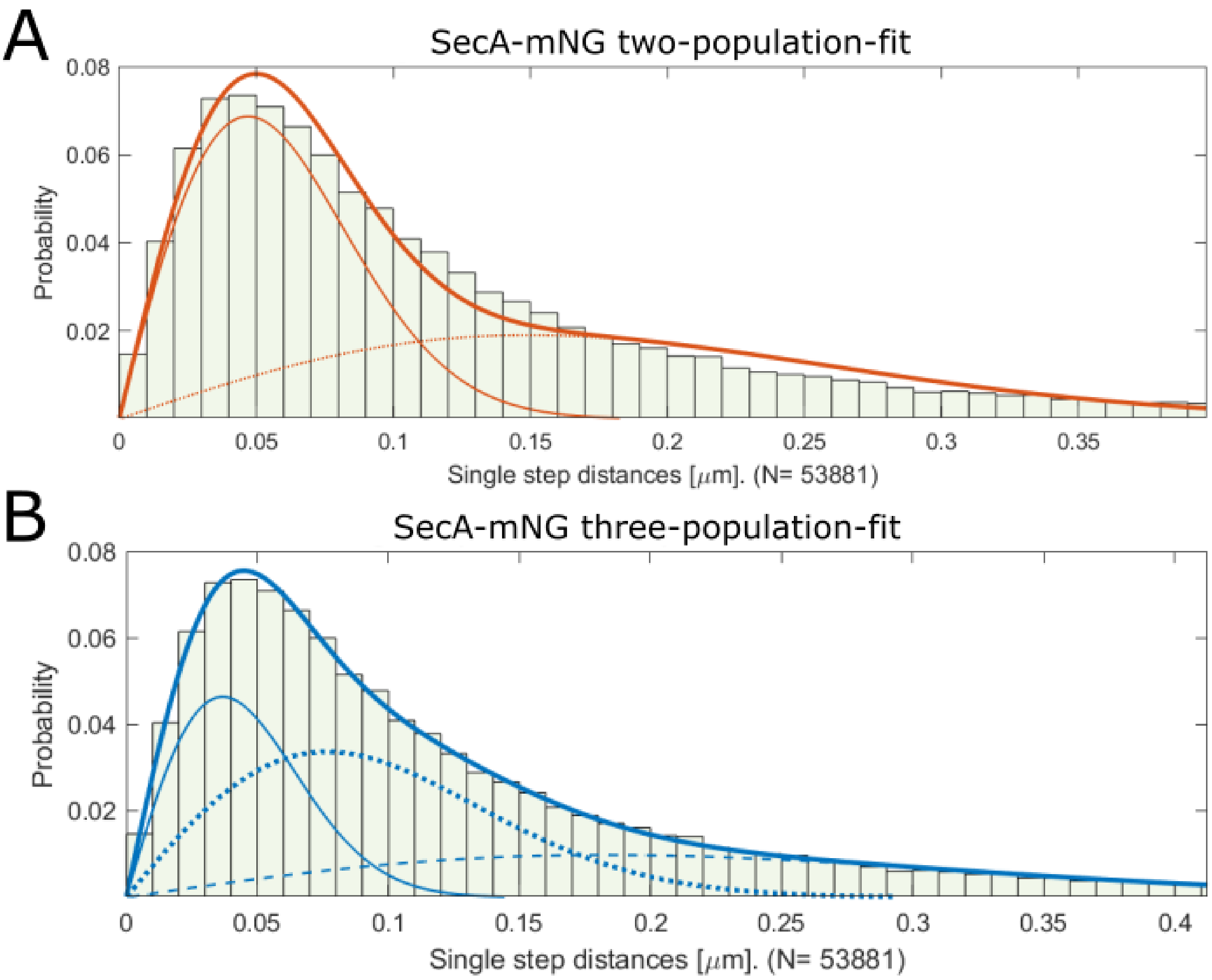

Figure S4: Comparison of a two- and a three-population fit of SecA-mNG at OD=2. Thick solid lines are combined with two – or three population fits. A) shows a two-population fit of the analysis of the jump distance analysis. The dashed line is the fast-mobile population, and the thin solid line is the slow-mobile population. B) shows the three-population fit. The thin solid line is the slow-mobile population, the dashed line is the medium-fast population, and the dotted line is the fast-mobile fraction.

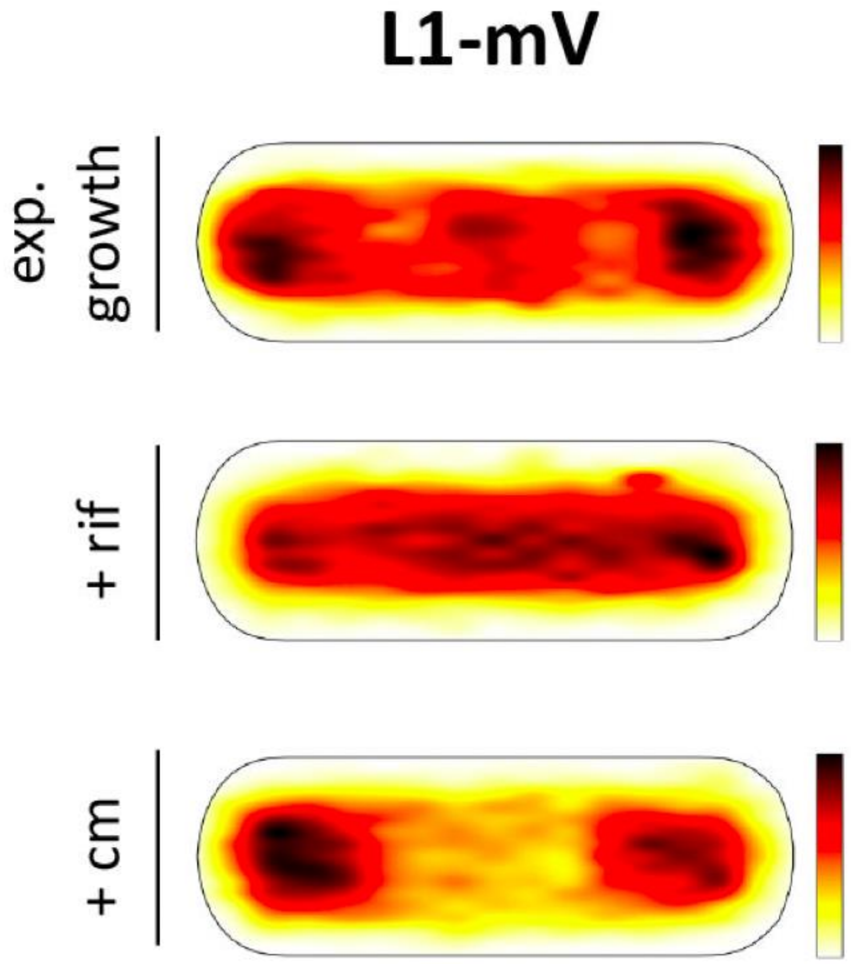

Figure S5 Heat maps generated from single molecule tracking of L1-mVenus (L1-mV) in *B. subtilis* cells, exponential growth phase (upper panel), 30 min after addition of Rifampicin (middle panel) or 30 min after addition of chloramphenicol (lower panel). Data set was taken from Stoll et al., 2022, Front. Microbiol. 13 doi: 10.3389/fmicb.2022.999176.

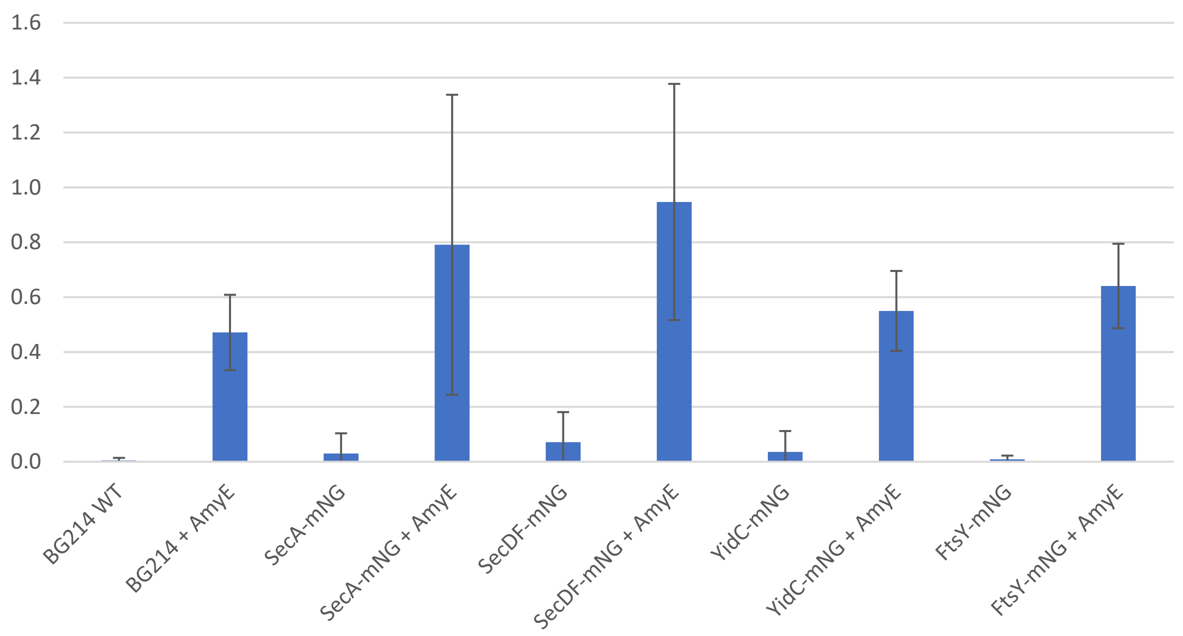

Figure S6: Comparison of the AmyE-overexpression strains with the strains with wildtype level of AmyE. For the investigation, a phadebas assay was used. All strains are based on *Bacillus subtilis* BG214. Therefore, the wildtype BG214 and BG214 with the overproduction plasmid are the positive and negative controls. All strains called with AmyE contain the overproduction plasmid pm11k_amyEBs.

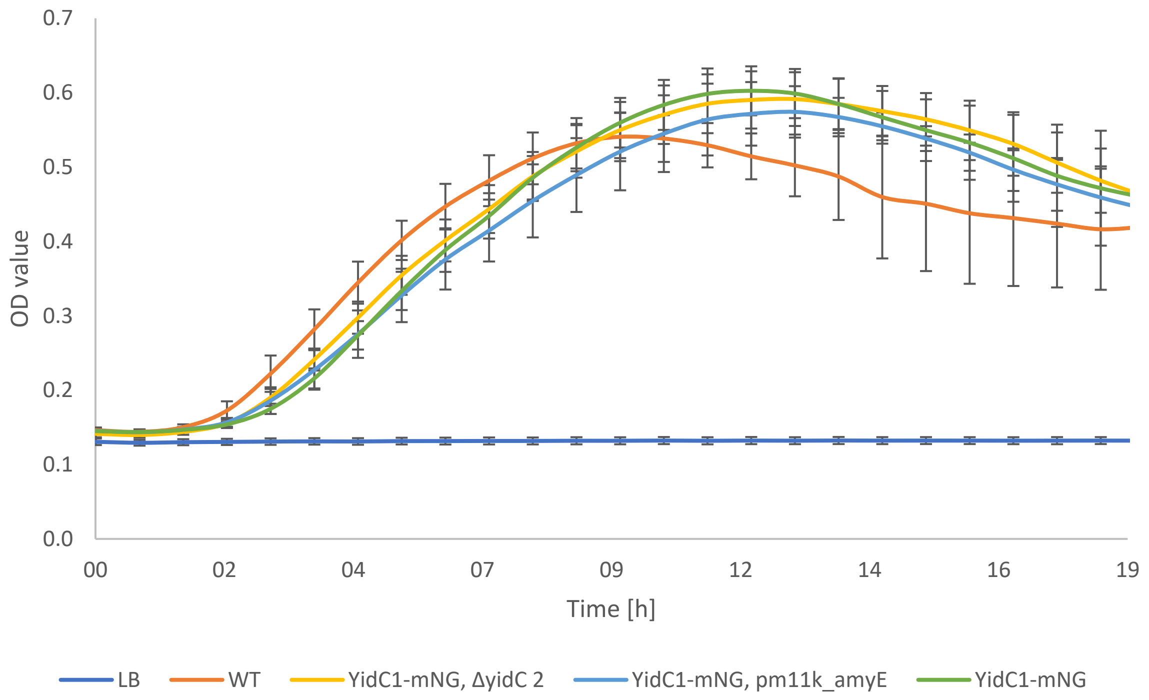

Figure S7: Growth curve of YidC-mNG in different backgrounds as a functionality test. It is known that deleting yidC1 and yidC2 is lethal (Kumazaki, et al. 2014). LB and the wildtype (WT) are negative and positive controls. YidC1-mNG (green) is the strain tracked. YidC1-mNG, pm11k_amyE contains the overproduction plasmid of AmyE. This strain was also tracked. YidC1-mNG, ΔyidC2 should only be growing with a functional YidC1. This can be shown here.

Table S1: Strains used in this work.

**Number**	**Strain name**	**Relevant features**
PG3167	*Bacillus subtilis* BG214	Wildtype (base for all other strains)
PG4615	SecA-mNG	*secA-mNeonGreen*^{cmR}
PG4614	SecA-mNG AmyE	*secA-mNeonGreen*^{cmR}, pM11K_amyEBs
PG4612	SecDF-mNG	*secDF-mNeonGreen*^{cmR}
PG4613	SecDF-mNG AmyE	*secDF-mNeonGreen*^{cmR}, pM11K_amyEBs
PG4279	FtsY-mV	*ftsY-mVenus*^{cmR}
PG4618	FtsY-mV AmyE	*ftsY-mVenus*^{cmR}, pM11K_amyEBs
PG4605	YidC1-mNG	*yidC1-mNeonGreen*^{cmR}
PG4606	YidC1-mNG AmyE	*yidC1-mNeonGreen*^{cmR}, pM11K_amyEBs
PG4616	YidC1-mNG, yidC2	*yidC1-mNeonGreen*^{cmR}, Δ*yidC2::kan trpC2*

Table S2: General data for the single molecule tracking.

Figure	Condition	# movies	# cells	# tracks	#tracks/cell
Figure 1	SecA-mNG OD 2	15	75	6080	89.11
	SecA-mNG AmyE OD 2	9	62	5980	97.28
	SecA-mNG OD 4	9	58	6699	112.90
	SecA-mNG AmyE OD 4	8	40	6743	168.1
	SecA-mNG OD 6	8	72	5712	88.81
	SecA-mNG AmyE OD 6	6	73	5879	87.11
Figure 2	SecA-mNG	22	142	9812	75.73
	SecA-mNG Cm	30	207	10965	56.76
	SecA-mNG Rif	13	119	10443	95.25
Figure 3	SecDF-mNG	15	90	2472	28.96
	SecDF-mNG AmyE	16	103	2543	24.68
Figure 4	FtsY-mV	6	52	1250	25.76
	FtsY-mV AmyE	15	91	1075	13.25
Figure 5	YidC-mNG	13	113	5346	50.14
	YidC-mNG AmyE	12	102	5216	53.70
